# Choice of Antiplatelet vs. Anticoagulant for Blunt Cerebrovascular Injury

**DOI:** 10.3390/life15091364

**Published:** 2025-08-28

**Authors:** Denise Baloi, Yusor Al-Nuaimy, Ammar Saloum, Elham Rahmanipour, Mohammad Ghorbani, Michael Karsy, Brandon Lucke-Wold, Mehrdad Pahlevani

**Affiliations:** 1 College of Human Medicine, Michigan State University, Lansing, MI 48823, USA; baloiden@msu.edu (D.B.); ammarsaloum18@gmail.com (A.S.); 2 College of Medicine, Ajman University, Ajman 24404, United Arab Emirates; amjdnuaimy@gmail.com; 3 Immunology Research Center, Mashhad University of Medical Sciences, Mashhad 91779-48564, Iran; elhamrahmanipour@gmail.com; 4 Department of Orthopaedics Surgery, Orthopedic Research Center, Mashhad University of Medical Sciences, Mashhad 91779-48564, Iran; ghorbani.m@aol.com; 5 Department of Neurosurgery, University of Michigan, Ann Arbor, MI 48105, USA; mkarsy@med.umich.edu; 6 Department of Neurosurgery, University of Florida, Gainesville, FL 32608, USA; brandon.lucke-wold@neurosurgery.ufl.edu; 7 Department of Neurosurgery, Jamaica Hospital Medical Center, New York City, NY 11418, USA

**Keywords:** blunt cerebrovascular injury, BCVI, antiplatelet therapy, anticoagulant therapy, antiplatelet versus anticoagulant

## Abstract

Blunt cerebrovascular injury (BCVI) defines a relatively common finding in trauma-injured patients, seen in roughly 2.4% of trauma patients. Unrecognized and untreated complications of BCVI can include stroke and neurological deficits. Increased use of CT angiography has led to a greater incidence of BCVI in traumatic brain injury and polytrauma patients, prompting a greater understanding of treatment options to mitigate morbidity. Antiplatelet medications, including aspirin and P2Y12 inhibitors (e.g., clopidogrel), as well as warfarin, dual oral anticoagulants (DOAC, e.g., dabigatran, rivaroxaban, apixaban, and edoxaban) provide a wide variety of medical treatment options. This article serves as a review of current evidence from 2015 to 2025 regarding best practices involving antiplatelet and anticoagulation use in the treatment of BCVI.

## 1. Introduction

Blunt cerebrovascular injury (BCVI) includes traumatic injuries to the internal carotid artery (ICA) or vertebral artery (VA), and is identified in approximately 2.4% of admitted trauma patients [1,2]. Serious complications of BCVI include stroke and disability [3,4,5]. In an early case series, the overall mortality has been reported to be 59% in patients with BCVI, of which 80% of those were directly linked to BCVI [6], and treatments aim to reduce the occurrence [1,4]. The most common mechanisms of BCVI include extreme cervical hyperextension or rotation, direct blunt vascular trauma, trauma to the intraoral region, and direct lacerations from bony fragments secondary to fractures such as cervical vertebrae or the skull base [7].

There remains ongoing debate regarding whether antiplatelet agents or anticoagulants offer the optimal therapeutic approach for BCVI, regarding reducing stroke risk and bleeding complications [3]. A national survey of clinical management practices reflected this variability: 43% of respondents preferred anticoagulation, 32.5% used antiplatelet therapy, 17% employed both, and 7.5% opted for procedural interventions [8]. Some limitations in effective guidelines include the large heterogeneity in included BCVI patients, the lack of high-quality data to make treatment decisions, and the rarity of delayed stroke events following BCVI. To better understand the concepts of ischemic adverse events after BCVI and employ the optimal and on-time treatment modality to prevent short-term and long-term complications, morbidity, and mortality, a deep dive into the current diverse neurotrauma literature is deemed practical and useful for clinicians. We performed a narrative review comparing the efficacy of antiplatelet and anticoagulant therapies in the management of BCVI. Outcomes such as stroke prevention, hemorrhagic complications, and overall clinical effectiveness were compared to help guide future clinical decision-making and identify areas in need of further research.

## 2. Materials and Methods

A literature search was conducted in English-language articles for the past 10 years (2015–2025). PubMed, Scopus, and Google Scholar databases for clinical trials, systematic reviews, meta-analyses, cohort studies, and case series. The search strategy used MeSH terms and keywords: “blunt cerebrovascular injury,” “BCVI,” “antiplatelet therapy,” “anticoagulant therapy,” “antiplatelet versus anticoagulant,” “prophylactic antithrombotic therapy,” “therapeutic antithrombotic therapy,” “antithrombotic medications,” “mechanism of action,” “clinical indications,” “timing of administration,” “duration of administration,” “route of administration,” “clinical outcomes,” “prognosis,” “thromboembolic complications,” “hemorrhagic complications,” and “complication management”. Trials were eligible if they reported on BCVI, antiplatelet or anticoagulant drug use, and clinical outcomes (See Table 1). Conference abstracts, non-peer-reviewed journals, and studies unrelated to the established goals were excluded.

**Figure 1:**
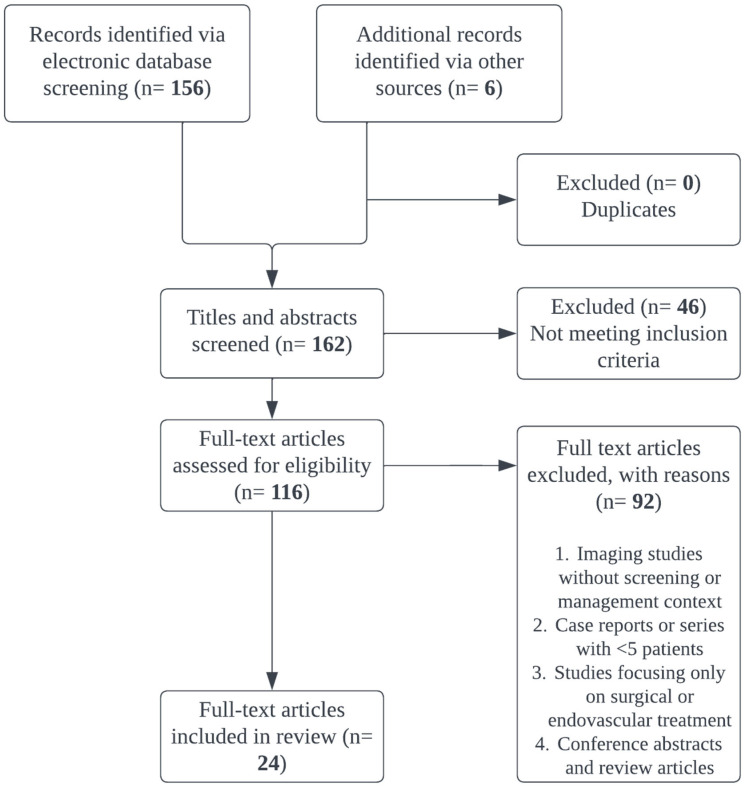
PRISMA Diagram for Systematic Review.

Articles were independently screened for titles and abstracts by at least 2 authors, and a 3rd author served as a tie-breaker. Full texts of selected articles were reviewed to extract relevant data, including study design, patient population, treatment modality, timing, outcome measures, and reported complications. Given the heterogeneity in study designs and outcome reporting, results were summarized qualitatively and organized according to key themes aligned with the review’s objectives. A total of 156 articles were identified and narrowed to 24 articles in the final analysis (See Figure 1).

## 3. Discussion

### 3.1. Overview of BCVI

**Figure 2:**
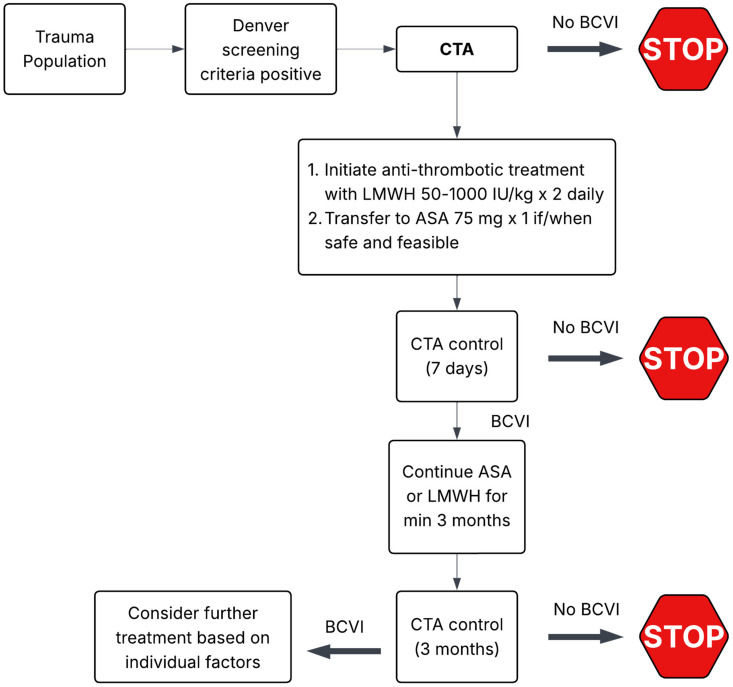
Summary of BCVI management [11].

Biffl et al. introduced a grading scale for BVCI to better guide prognosis and management of these cases [31]. The scale classified injuries from Grades I through V based on the conventional angiographic findings (See Table 2). Alsheik et al. further proposed that grade V injuries should be separated into either non-contained rupture with active extravasation or intravascular rupture, such as in the case of arteriovenous fistulas [32]. According to the grade of injury, the treatment could range from antithrombotic therapy for lower-grade injuries to endovascular therapies for higher-grade injuries [10,33,34] (See Figure 2). The advent and widespread availability of CTA has mainly replaced DSA as the first-line screening imaging modality of choice for BCVI [35,36]. However, the sensitivity of DSA is significantly higher than CTA [16,37,38]. MRI shows a sensitivity and specificity of 50–75% and 67% for detecting BCVI, and while it can be used to evaluate acute cerebral infarction and vessel wall abnormalities, it remains limited as a screening tool [39].

Prognostically, BCVI is an independent predictor for worse outcomes associated with both higher morbidity and mortality rates for those patients suffering from a stroke [2,10,40]. The reported complication rate is as high as 25–50% [2] In addition, a large number of BCVI patients may have developed a stroke by the time of initial presentation to the hospital. The reported range of ischemic stroke among BCVI patients on admission is 44–82% [41]. The true incidence of ischemic strokes post BCVI remains largely unknown, with the reported incidence in the literature ranging from 1 to 26% [2,10,29,42].

The risk of developing a stroke is tightly related to the grade of BCVI. Biffl et al. reported that the risk of stroke was greater with every increasing grade, namely an incidence of 3%, 11%, 33%, and 44% for grades I, II, III, and IV, respectively [14,36]. Moreover, the risk of stroke with grade IV BCVI is dependent on the site and type of occlusion: 9–20% risk of developing a stroke in the setting of unilateral vertebral artery occlusion, more than 50% with unilateral ICA occlusion, and 50% with bilateral vertebral artery occlusion [41].

The presence of hemorrhagic injury in 25–40% of BCVI patients is a therapeutic dilemma [30]. According to Callcut et al., the reported risk of increasing hemorrhagic injury in patients with traumatic brain injury (TBI) was not related to treatment with therapeutic heparin or aspirin [43]. These findings were confirmed by more recent studies [18,30,44,45]. As such, antiplatelet and antithrombotic therapy remain the mainstay for prevention of ischemic-related morbidity and mortality, albeit with dose adjustments being advised [46,47].

### 3.2. Medical Management of BCVI

**Figure 3:**
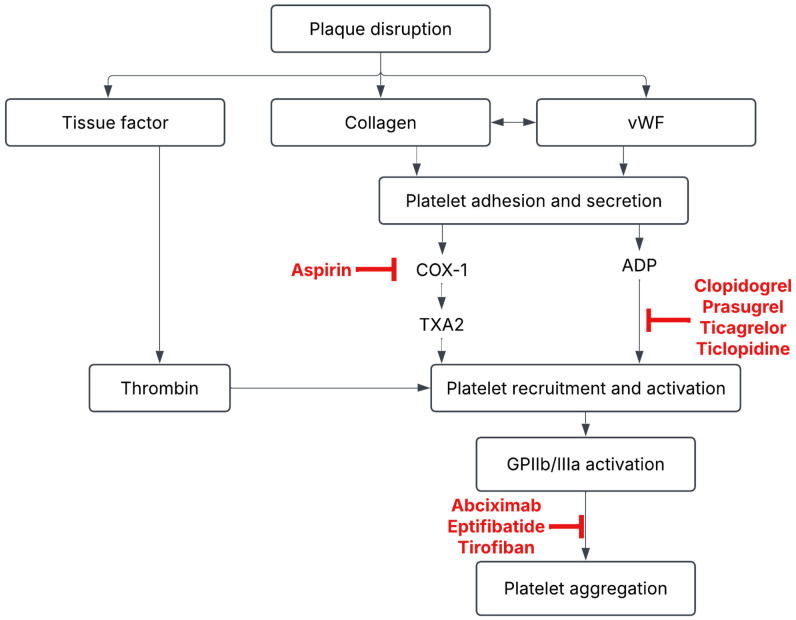
Antiplatelet cascade.

Antiplatelet agents are crucial in the prevention of arterial thrombosis, more so in patients at a high risk of developing stroke or cardiovascular events [24]. The most widely used antiplatelet, Aspirin (ASA), is an irreversible inactivator of cyclooxygenase-1 (COX-1) [48], leading to suppression of thromboxane A_2_ synthesis and inhibiting platelet aggregation (See Figure 3). It has been used as monotherapy in low-grade BCVI (grades I and II), with a generally favorable safety and efficacy profile [22]. Clopidogrel is a P2Y12 receptor antagonist which inhibits platelet activation via a distinct mechanism. Clopidogrel is often used as an alternative for patients who do not tolerate aspirin well or used in combination therapy (e.g., dual antiplatelet therapy) [27,49]. In the context of BCVI, treatment is usually continued for 3–6 months, with follow-up imaging [10,19]. As of current, guidelines from WTA and EAST recommend antithrombotic therapy, endovascular or surgical management, depending on the location and grade/severity of the injury. Recommendations from Brommeland et al. endorse that LMWH in antithrombotic doses should be initiated within 24–48 h of diagnosis, followed by a 75 mg dose of daily aspirin [10].

**Figure 4:**
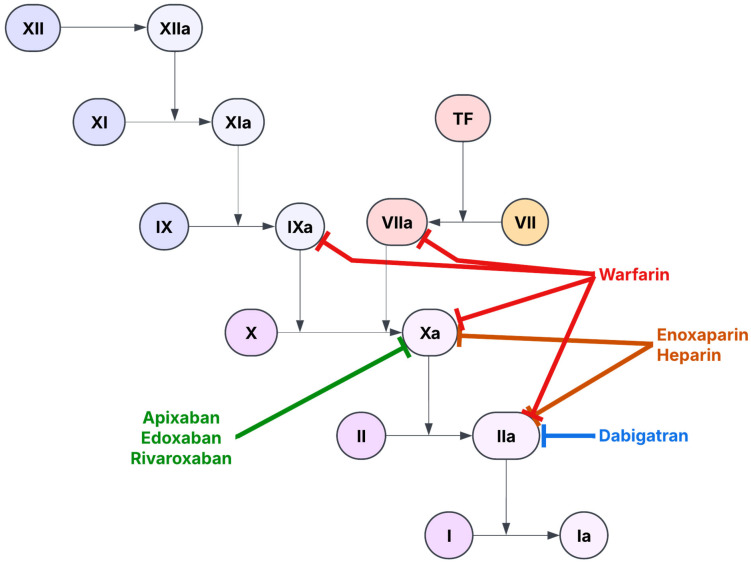
Anticoagulation cascade.

Anticoagulants are also used in thromboprophylaxis in patients with BCVI (See Figure 4) [50,51]. Unfractionated heparin (UFH) is typically the agent of choice in acute settings due to its rapid onset of action, relatively short half-life, and quick reversibility with protamine sulfate [26,52]. UFH works by forming a complex with antithrombin, a natural inhibitor of several activated clotting factors. This complex formation induces a conformational change that greatly accelerates Antithrombin’s ability to clear and inactivate thrombin and other prothrombotic factors. Low molecular weight heparin (LMWH) is a more “stable” treatment modality that can be used during the transition from inpatient to outpatient care [18]. Warfarin is a potent vitamin K antagonist which has been historically used for long-term anticoagulation. However, its narrow therapeutic index, need to monitor INR frequently, and extensive drug–drug and food-drug interactions have limited its use in favor of newer anticoagulant agents [53]. Direct oral anticoagulants (DOACs), such as dabigatran, rivaroxaban, apixaban, and edoxaban, have recently gained traction due to their convenient use (e.g., fixed dose, no routine monitoring, and lower risk of intracranial bleeding). Yet, evidence supporting their use specifically in BCVI cases is limited [54]. Currently, anticoagulation is typically reserved for BCVI grades II–IV. Despite their risk of hemorrhage, the growing body of literature supports prompt initiation of anticoagulants in BCVI [3].

### 3.3. Comparison of Anticoagulation and Antiplatelet

While the American Heart Association (AHA) and American Stroke Association (ASA) have not issued guidelines specifically addressing BCVI, their broader recommendations on stroke prevention can be applied to BCVI care [41]. The ASA guidelines advocate for antithrombotic therapy in patients at risk for cerebrovascular events tailored to etiology. For instance, antiplatelet therapy is preferred in patients with severe intracranial stenosis in ischemic stroke or transient ischemic attack (TIA), whereas anticoagulation is recommended for patients with atrial fibrillation [41]. Similarly, EAST supports early antithrombotic therapy in BCVI, though it does not specify a clear preference for antiplatelet versus anticoagulant agents [25,55,56]. While both EAST and AHA/ASA emphasize the importance of stroke prevention, there remains no consensus on what is optimal for BCVI [18]. Studies suggest that antiplatelet therapy may be as effective as anticoagulation in reducing stroke risk, while also potentially lowering the risk of bleeding complications [41,56]. Ultimately, the choice of antithrombotic therapy is often guided by individual patient determinants [57].

The optimal treatment for asymptomatic BCVI remains a subject of ongoing debate [58,59]. Systemic anticoagulation with heparin is the treatment of choice due to neurological improvement; however, the use of antiplatelet agents gained traction due to easier administration and a favorable safety profile [3,9]. Evidence supports the initiation of antithrombotic therapy in asymptomatic cases, as untreated patients have shown significant rates of complications [9,60]. A 2024 systematic review analyzing over 6500 patients found that antiplatelet therapy was associated with a lower risk of stroke compared to anticoagulation (OR 0.57; 95% CI 0.33–0.96; *p* = 0.04), along with a reduced risk of bleeding complications (OR 0.29; 95% CI 0.13–0.63; *p* = 0.002) [24]. Notably, five studies specifically comparing aspirin to heparin showed lower bleeding rates with aspirin (OR 0.16; *p* = 0.005) [24]. Antiplatelet therapy has been associated with lower six-month readmission rates compared to anticoagulants, with similar rates of cerebrovascular events and complications [17,19,61].

Contrastingly, while some analyses report comparable ischemic stroke rates between antiplatelet and anticoagulant therapies [9,60], others highlight potential advantages of anticoagulation [19]. In a study of the Nationwide Readmission Database between 2011 and 2025, a comparison of 725 BCVI patients showed a lower post-discharge rate of cerebrovascular accidents (1.8% vs. 5.72%) and mortality (1.3% vs. 4.9%) with anticoagulation compared with antiplatelet use [16]. This series showed the majority of patients (69%) demonstrating Biffle grade 3 injury [16], suggesting the potential benefit for anticoagulation in higher grade injuries over antiplatelet.

High-grade BCVI, including occlusions (Grade IV) and transections (Grade V), also pose treatment challenges due to their severity and the high risk of injury [20]. Antithrombotic therapy remains the key for management, as it is consistently linked to a reduced incidence of adverse neurologic outcomes across all BCVI grades [20]. A recent analysis using the Nationwide Readmission Database found no significant difference between antiplatelet and anticoagulant therapies in terms of index mortality, six-month mortality, or six-month cerebrovascular accident (CVA) readmissions [16]. However, patients on anticoagulants had higher six-month readmission rates. There was no significant difference in hemorrhagic CVA and gastrointestinal bleeding between antiplatelet and anticoagulation.

Existing data suggest that antiplatelets may offer comparable protection against stroke while potentially reducing the risk of hemorrhagic complications [21]. A systematic review found similar stroke rates between patients treated with antiplatelets and those receiving anticoagulation, but with a significantly lower incidence of bleeding in the antiplatelet group (2% vs. 6%) [23]. This trend highlights the potential advantage of antiplatelet therapy, especially in patients where bleeding risk is a concern. An important consideration is that patients with BCVI frequently present with additional traumatic injuries such as traumatic brain injury, spinal trauma, or solid organ damage that may initially limit the use of antithrombotic therapy. However, evidence suggests that antithrombotic treatment can still be safe in these settings [21].

### 3.4. Pediatric BCVI Treatment

The management of BCVI in children remains challenging due to the scarcity of pediatric-specific data, often requiring consultation of adult treatment guidelines. A multicenter retrospective cohort study by Ravindra et al. (2021) examined outcomes in children under 10 years old and found no significant differences in hemorrhagic complications between those treated with antiplatelet versus anticoagulant therapy [26]. Interestingly, there was a nonsignificant trend toward better vessel healing in the antiplatelet group, suggesting a potential benefit in younger patients [23,62]. Despite these findings, a recent commentary emphasized the need for further high-quality studies before making definitive changes to clinical practice, highlighting the uncertainty that still surrounds this age group [28].

### 3.5. Elderly BCVI Treatment

Elderly patients with BCVI pose a complex dilemma due to increased susceptibility to thrombosis and hemorrhage, compounded by comorbidities and use of other medications [54]. Age-related vascular fragility and diminished renal function can elevate the bleeding risk associated with anticoagulant therapy [54]. Data from the literature indicates that the risk of major bleeding is comparable between antiplatelet and anticoagulant treatments in this population, suggesting that the decision should be individualized to the patient [11]. Furthermore, the Japan Geriatrics Society has cautioned against the overuse of multiple antithrombotic agents in older adults due to the potential for adverse drug interactions and poor adherence [54]. Xa inhibitors are currently recommended for elderly patients initiating anticoagulation, particularly when renal function is preserved [54]. In summary, while both therapies may be effective, antiplatelet agents might be a safer alternative.

### 3.6. Polytrauma BCVI Treatment

In polytrauma patients, the risks and benefits of antithrombotic therapy merit significant consideration. Data from the Prospective Vascular Injury Treatment registry emphasized the importance of administering some form of antithrombotic therapy, either antiplatelet or anticoagulant, to reduce the risk of stroke and death, while highlighting the dangers of withholding treatment altogether [27]. A 2021 meta-analysis of over 2000 BCVI patients found no difference in ischemic stroke rates between antiplatelet and anticoagulant therapies but reported a lower hemorrhage rate in those receiving antiplatelets, suggesting a safety advantage in trauma populations [26]. In cases of concomitant traumatic brain injury (TBI), anticoagulant use has been linked to a higher risk of significant intracranial hemorrhage, particularly with agents like warfarin, while aspirin did not show this elevated risk [26,53]. Collectively, these findings suggest that antiplatelet therapy may be the preferred option in polytrauma patients, especially when intracranial injuries are present or bleeding risk is elevated.

## 4. Conclusions

Although treatment with anticoagulants and antiplatelet agents exposes blunt head trauma patients to increased risk of hemorrhagic complications, the decision to start each therapy should be individualized. Data supports the use of anticoagulation in higher-grade injury patterns (Biffl 3 or higher); however, supportive data are limited. More prospective studies are needed to strongly recommend the use of anticoagulants or antiplatelet agents in equivocal situations and in clinical scenarios where making appropriate decisions is accompanied by changes in morbidity and mortality. Consideration of other patient comorbidities may be helpful for treatment. Anticoagulation may be preferred in the setting of concomitant cardiac arrhythmia or deep vein thrombosis, while antiplatelets may be more helpful in secondary stroke prevention or high-risk hemorrhage situations.

## Figures and Tables

**Table 1:** Studies evaluating anticoagulation and antiplatelet medications in BCVI.

Reference	Sample Size	Anticoagulation Use	Outcomes Evaluated	Morbidity	Mortality	Mean Follow-Up
Barrera et al. 2020 [9]	189 cases	Yes	Ischemic stroke, blood transfusion	VAI stroke rate: 55.3% CAI stroke rate: 30.9% Stroke rate of both: 13.8%	5.80%	N/A
Brommeland et al. 2018 [10]	78 studies	Yes	Radiological investigation, vessel injury, stroke, morbidity, mortality	N/A	N/A	N/A
Bonow et al. 2021 [11]	677 cases	Yes	Stroke after admission	Overall stroke rate: 8.11%	Overall: 10.2%	N/A
Burlew et al. 2018 [4]	492 cases	Yes	Time to stroke	N/A–cohort is stroke-focused	Overall: 32%	N/A
Covino et al. 2021 [12]	685 cases	Yes	Delayed intracranial hemorrhage	N/A	N/A	N/A
Coyle et al. 2024 [13]	8 studies	Yes	Intracranial bleeding	N/A	N/A	N/A
Esnault et al. 2017 [14]	228 cases	Yes	Neurologic outcomes	Overall stroke rate: 16%	Overall: 33%	95.2% of patients had clinical follow-up at 3 months
Figueroa et al. 2021 [15]	30 cases	Yes	Recovery, short-term outcomes (hospitalization), long-term outcomes (speech and language deficits)	Overall stroke rate: 23.7%	Overall: 7.9%	68% patients had clinical f/u of which 8 patients had f/u arteriography
Hanna et al. 2020 [16]	725 cases	Yes	Rate of cerebrovascular accident post-discharge	On anticoagulants: 1.8% On antiplatelets: 5.72%	On anticoagulants: 4.2% On antiplatelets: 3.9%	N/A
Hiatt et al. 2023 [17]	77 cases	Yes	Neurologic deficits on follow-up, emboli on transcranial Doppler ultrasound, new or worsening intracranial hemorrhage, extracranial bleeding complications, ischemic stroke, death	Overall stroke rate: 3.5%	Overall: 20.9%	76.02% patients had at least one f/u arteriography
Kim et al. 2020 [18]	10 studies	Yes	Stroke incidence, hemorrhagic deterioration	N/A	N/A	N/A
Ku et al. 2021 [19]	2044 cases	Yes	Ischemic stroke rate, hemorrhagic complications	N/A	N/A	N/A
Lau et al. 2024 [20]	11 cases	Yes	Stroke, mortality	Overall stroke rate: 31.6%	Overall: 29.8%	N/A
Lauerman et al. 2015 [21]	82 cases	Yes	Stroke after admission	CAI GIV stroke rate: 70% VAI GIV stroke rate: 2.9%	CAI: 38.5% VAI: 5.8%	N/A
Leverich et al. 2023 [22]	1091 cases	Yes	Morbidity, mortality, readmission rate	Overall (ischemic) stroke rate: 2.9% Overall (hemorrhagic) stroke rate: 2.4%	Overall: 2.1%	N/A
Matsushima et al. 2020 [23]	168 cases	Yes	Traumatic brain injury progression	N/A	N/A	N/A
Momic et al. 2024 [24]	7643 cases	Yes	Stroke risk, bleeding complications	N/A	N/A	N/A
Probst et al. 2019 [25]	9070 cases	Yes	Intracranial bleeding	N/A	N/A	N/A
Ravindra et al. 2020 [26]	18 cases	Yes	Hemorrhagic complications, healing	Overall stroke rate: 47.05%	Overall: 5.8%	82.5% patients had f/u arteriography
Russo et al. 2021 [27]	971 cases	Yes	Stroke, mortality	Overall stroke rate: 7%	Overall: 12%	N/A
Shafafy et al. 2017 [28]	258 BCVI cases, 23 asymptomatic	Yes	Neurological outcomes, grade of injury, death	N/A	N/A	N/A
Shahan et al. 2016 [29]	119 cases	Yes	Morbidity, mortality	Overall stroke rate: 9%	Overall: 9.7%	N/A
Hundersmarck et al. 2021 [5]	71 cases	No, antiplatelet only	In-hospital stroke	Overall stroke rate: 28%	Overall: 23%	83% patients had f/u arteriography
Tso et al. 2017 [30]	3049 cases	No, antiplatelet only	Ischemic complications	Overall stroke rate: 29.1%	Overall: 8%	N/A

**Table 2:** Biffl Scale for Blunt Cerebrovascular Injury.

Grade Injury	Angiographic Characteristics
Grade I	Non-hemodynamically significant intramural irregularity with 25% luminal narrowing
Grade II	Potentially hemodynamically significant dissections and hematomas with 50% luminal narrowing presence of intraluminal thrombus, or a dissection with a visible raised intimal flap
Grade III	Pseudoaneurysms
Grade IV	Occlusions
Grade V	Vessel transections with active extravasation

## Data Availability

No new data was created or analyzed in this study. Data sharing is not applicable to this article.

## Abbreviations

The following abbreviations are used in this manuscript:

BCVI	Blunt Cerebrovascular Injury
DOAC	Dual Oral Anticoagulants
ICA	Internal Carotid Artery
TBI	Traumatic Brain Injury
CCF	Carotid-Cavernous Fistulas
WTA	Western Trauma Association
EAST	Eastern Association for the Surgery of Trauma
CTA	Computed Tomography Angiography
MRA	Magnetic Resonance Angiography
DSA	Digital Subtraction Angiography
ASA	Aspirin
COX	Cyclooxygenase
UFH	Unfractionated Heparin
LMWH	Low Molecular Weight Heparin
AHA/ASA	American Heart Association/American Stroke Association
CVA	Cerebrovascular Accident
